# The olfactory hole-board test in rats: a new paradigm to study aversion and preferences to odors

**DOI:** 10.3389/fnbeh.2015.00223

**Published:** 2015-08-25

**Authors:** Kerstin E. A. Wernecke, Markus Fendt

**Affiliations:** ^1^Institute for Pharmacology and Toxicology, Otto-von-Guericke University MagdeburgMagdeburg, Germany; ^2^Center for Behavioral Brain SciencesMagdeburg, Germany

**Keywords:** approach, avoidance, buspirone, carnivore urine, innate fear, female rat urine, hole-board test, predator

## Abstract

Odors of biological relevance (e.g., predator odors, sex odors) are known to effectively influence basic survival needs of rodents such as anti-predatory defensiveness and mating behaviors. Research focused on the effects of these odors on rats’ behavior mostly includes multi-trial paradigms where animals experience single odor exposures in subsequent, separated experimental sessions. In the present study, we introduce a modification of the olfactory hole-board test that allows studying the effects of different odors on rats’ behavior within single trials. First, we demonstrated that the corner holes of the hole-board were preferentially visited by rats. The placement of different odors under the corner holes changed this hole preference. We showed that holes with carnivore urine samples were avoided, while corner holes with female rat urine samples were preferred. Furthermore, corner holes with urine samples from a carnivore, herbivore, and omnivore were differentially visited indicating that rats can discriminate these odors. To test whether anxiolytic treatment specifically modulates the avoidance of carnivore urine holes, we treated rats with buspirone. Buspirone treatment completely abolished the avoidance of carnivore urine holes. Taken together, our findings indicate that the olfactory hole-board test is a valuable tool for measuring avoidance and preference responses to biologically relevant odors.

## General Introduction

Challenged by the large diversity of natural odor blends in their environment, most mammalian species have developed highly sensitive olfactory systems to identify and discriminate biologically relevant odors. In rodents, the detection of some odors is of critical importance because they trigger different basic behaviors essential for their survival. In particular, odors transmitted between individuals of the same species (pheromones) are used to communicate information on the gender, reproductive state, social status, and subject identity. Thus, pheromones have been highly associated with the mediation of, e.g., mate choice, parental care and territorial behaviors (Brennan and Kendrick, 2006; Fortes-Marco et al., 2013). Predator odors present a different group of biologically relevant odors called kairomones (Fortes-Marco et al., 2013; Rajchard, 2013). Kairomones are odors that damage the interests of the releaser while being beneficial for the receiving animal (of another species). In this context, predator odors warn prey animals of a potential confrontation with a predator. For example, odors derived from cats or other carnivorous species (e.g., urine samples from foxes, bobcats, pumas, and coyotes) elicit a range of innate defensive behaviors in rodents including avoidance and hiding behavior, an increase in risk assessment behaviors and the suppression of non-defensive behaviors such as foraging, sexual behavior and overall locomotor activity (Blanchard and Blanchard, 1989; Apfelbach et al., 2005; Endres et al., 2005; Masini et al., 2005; Fendt, 2006; Wernecke et al., 2015).

Research focused on the effects of odors on rats’ behaviors often include multi-trial paradigms where animals experience a sequence of single odor exposures (e.g., Wallace and Rosen, 2000; Fendt, 2006; Ferrero et al., 2011; Rivard et al., 2014). In the current set of experiments, we have used a modified version of the olfactory hole-board test (Moy et al., 2008) to study behavioral effects of different odors within single trials. In this procedure, rats are placed in a standard hole-board apparatus with automated recording of nose pokes, also called head dips. Previous work has shown that the hole-board test offers a simple method for measuring exploratory behavior of animals in an unfamiliar environment (Takeda et al., 1998; Brown and Nemes, 2008). Whether an animal prefers or avoids a hole results from an inner conflict between the natural drive of rodents to explore and the potential aversive properties of the hole. Thus, according to this hypothesis, a general decrease in head dipping behavior is interpreted to reflect increased anxiety in animals, while high levels of head dipping behavior are defined as a decline in anxiety (Crawley, 1985; Lister, 1990; Saitoh et al., 2006).

In the present study, a series of four experiments has been conducted to investigate if the hole-board test can be used to investigate behavioral responses of rats to different odors within single trials. In *Experiment 1*, rats were tested for hole preference in the classical 16-hole configuration. *Experiments 2* and *3* were conducted to assess whether rats display a shift in hole preference when both aversive and attractive odors were presented in the preferred corner holes. *Experiment 4* tested if avoidance behavior to holes with carnivore urine samples can be reduced by treating the rats with the anxiolytic compound buspirone.

## General Materials and Methods

### Subjects

Testing was carried out using 64 experimentally naive male Sprague-Dawley rats (2–3 months-old) weighing 200–350 g at the time of testing. Rats were bred and reared at the local animal facility (original breeding stock: Taconic, Denmark). They were housed in groups of 5–6 animals in standard Macrolon Type IV cages with water and food available *ad libitum*. Cages were kept in temperature and humidity-controlled rooms (22 ± 2°C, 50–55%) under a 12 h light/dark cycle with lights on at 6:00 am. Behavioral testing was conducted during the light phase between 8:00 am and 3:00 pm.

All experiments were carried out in accordance with international ethical guidelines for the care and use of laboratory animals for experiments (2010/63/EU), and were approved by the local authorities (Landesverwaltungsamt Sachsen-Anhalt, Az. 42505-2-1172 UniMD).

### Testing Apparatus

**FIGURE 1:**
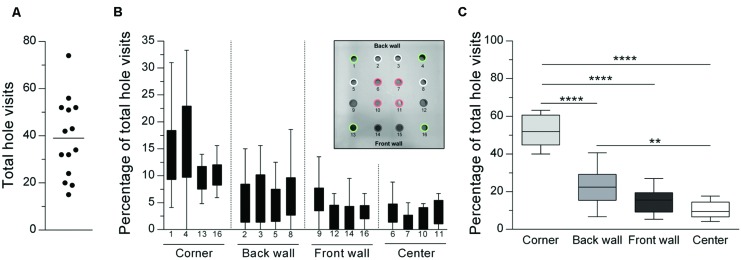
**General distribution of hole visits (median) during a hole-board test showing a clear preference for corner holes. (A)** Total numbers of hole visits for individual animals (*n* = 14) are displayed as a scatter dot plot with the horizontal line describing the mean. Number of hole visits (% of total) are depicted for **(B)** all 16 holes and **(C)** summed up for each hole category. ^∗∗^*p* < 0.01; ^∗∗∗∗^*p* < 0.0001 comparisons as indicated (Holm-Sidak’s multiple comparison test after significant main effects in an ANOVA).

All experiments were conducted in a computer-controlled hole-board apparatus (ActiMot2 Hole-Board System, TSE Systems, Bad Homburg, Germany) consisting of three testing boxes constructed from transparent Plexiglas (51.5 cm× 51.5 cm× 41 cm) and a height-adjustable frame with infrared detectors (sample rate: 100 Hz). A removable hole-board with 16 holes (3 cm diameter) in a grid-pattern was placed on the floor of the testing box. Holes were categorized into four corner holes (holes 1, 4, 13, 16; see **Figure 1B** Inlay), four back wall holes (holes 2, 3, 5, 8), four front wall holes (holes 9, 12, 14, 15) and four center holes (holes 6, 7, 10, 11). Supplier-specific lids were used to close particular holes, meaning that the number and/or the location of the holes could be modified as required for each experiment. The apparatus was located in a small testing room with dimmed illumination (illumination: ∼30 lx).

### General Testing Procedure and Odor Presentation

For *Experiment 1*, the hole-board was used in its 16-hole configuration. Rats were individually placed into the testing box and tested for 20 min. Rats head-dipping behavior was monitored by the infrared detectors. The software automatically measured the total number of head dips (hole visits) for each single hole. More specifically, a head dip was counted when the animal placed its head into a hole for at least 300 ms with the ears even with the floor of the hole-board. A minimal time interval of 300 ms had to elapse after a head dip before a new hole visit was counted. For the experiments with odor presentations (*Experiments 2–4*), the holes of the center region were covered (12-hole configuration). Then, animals were individually placed into the testing box and exposed to four odors simultaneously. For this, 1 ml odor samples (described in detail below) were pipetted into small glass bowls (4 cm outer diameter, 2.5 cm height) and placed underneath the hole of each corner prior to testing. The animals were not able to touch the odor samples. For each test session, one corner hole contained only water which served as a control odor. The location of the different types of odor samples was pseudo-randomly changed across individuals (*Experiments 2–4*) and tests (*Experiment 4*). The wall holes were always left empty. Head dips into the wall holes were used to assess baseline levels of exploration behavior and to control for individual differences in the total number of head dips. After each test, the testing boxes were thoroughly cleaned with soapy water and ventilated with clean air, before the next rats were tested.

### Odors

Urine samples from foxes, bobcats, pumas and coyotes were purchased from Maine Outdoor Solutions Inc., (Hermon, ME, USA). We previously demonstrated that urine samples of these carnivores induce avoidance behavior in an open-field experiment (Fendt, 2006). Urine samples from elks and mona monkeys were obtained from the local zoo (Zoologischer Garten Magdeburg, Magdeburg, Germany). Female rat urine was self-collected by placing adult female Sprague-Dawley rats (*n* = 12, 3–6 months-old) individually in a metabolic cage (Tecniplast, Hohenpeißenberg, Germany) for ∼30 min on consecutive days. Female urine samples of individual animals were mixed up to ensure that urine from all estrus cycle phases were present. All urine samples were aliquoted into 1 ml portions and stored at – 18°C until usage.

### Descriptive and Statistical Analysis

Hole visits were expressed as percentages of total hole visits. In all figures, behavioral data are shown as box-and-whisker plots. The horizontal line represents the median and the box the lower and upper quartiles. The whiskers were calculated with the Tukey method (GraphPad Prism 6.00, GraphPad Software Inc., La Jolla, CA, USA).

For statistical analysis, data were first tested on normal distribution (D’Agostino and Pearson omnibus test). For normally distributed data, analyses of variance (ANOVA) and *post hoc* comparisons by Holm-Sidak’s test were used. Non-normally distributed data were analyzed using the Friedman test followed by Dunn’s multiple comparisons test. Either hole location (*Experiment 1*) or odor (*Experiments 2–4*) was used as within-subject factor. A *p* < 0.05 was considered statistically significant. All analyses were carried out using GraphPad Prism.

Pilot tests revealed that the hole visit behavior of animals that are extremely active or extremely inactive is only marginally modulated by odors (floor/ceiling effects; see also discussion of *Experiment 1*). In *Experiments 2–4*, we therefore excluded animals with more than 65 or less than 15 total hole visits from further analysis.

## Experiment 1

The first experiment was conducted to determine whether rats display a specific exploration pattern in the hole-board test when no odors are present. From other exploration-based tasks (e.g., open-field) it is known that rodents prefer to remain in the periphery of the apparatus (thigmotaxis), whereas the bright and unprotected areas are usually avoided (Lister, 1990; Wallace and Rosen, 2000; Litvin et al., 2008). Therefore, we expected that our rats would show preference (i.e., a high number of visits) to the holes in the corners and along the side walls, and avoidance (i.e., a low number of visits) to the four holes in the center of the box. This was also observed in the mouse version of the olfactory hole-board test (Moy et al., 2008).

### Subjects and Procedure

Fourteen male Sprague-Dawley rats were tested. They were put into the middle of the hole-board (all 16 holes open, no odors) and their hole visits were recorded for 20 min.

### Results

**Figure 1** illustrates the local distribution of hole visits (16 holes, no odor). The total number of hole visits ranged between 15 and 74 head dips (**Figure 1A**), with a mean of 39 head dips. The subsequent analysis revealed that there were clear differences in the percentage number of total hole visits according to the position of the holes [corner vs. center vs. front wall vs. back wall: ANOVA: *F*_(3,39)_ = 66.21; *p* < 0.0001; **Figures 1B,C**]. Corner holes were visited significantly more often than holes with other locations (Holm-Sidak’s tests: *p*s < 0.0001). Furthermore, the back wall holes were visited more often than the center holes (*p* = 0.001). There were no significant differences in the hole visits within the different hole categories [corner holes: Friedman test: *Q* = 3.24; *p* = 0.36; wall holes: ANOVA: *F*_(7,91)_ = 2.21; *p* = 0.075; center holes: Friedman test: *Q* = 6.49; *p* = 0.09].

### Discussion

The behavior of animals in the hole-board test, originally described by Boisser and Simon (1962, 1964), is determined by a conflict between curiosity-based exploration and fear-based avoidance from novel, unknown locations (Hughes, 2007; Brown and Nemes, 2008). Thus, altered head dipping activity is often interpreted as changes in the anxiety state of the animals (Takeda et al., 1998; Brown and Nemes, 2008). In *Experiment 1*, rats were tested in the 16-hole configuration of the hole-board without any odors. Our results indicate that rats showed the highest rate of head dips for corner holes, and the lowest rate of head dips for center holes (**Figures 1B,C**). This finding is in line with the results of previous hole-board and related exploration-based rodent models and can be explained by thigmotaxis (Lister, 1990; Lamprea et al., 2008; Moy et al., 2008).

Interestingly, Moy et al. (2008) also established an olfactory hole-board test, however, to model repetitive behavior, a core symptom of autism, in mice. In their study, different appetitive odor samples (e.g., familiar cage bedding, food items) were presented in the less-preferred center holes and the ability of mice to shift their hole preference was assessed. In contrast to this, we wanted to mainly investigate how hole visits are influenced by aversive odors. Based on previous studies from our laboratory (Fendt, 2006; Ferrero et al., 2011; Wernecke et al., 2015), we expected that holes with aversive odors will be visited less often, i.e., avoided. Such avoidance is much easier to observe when holes are very often visited under control conditions. Therefore, our approach was to place the test odors under the corner holes. To further increase the number of corner hole visits, we closed the four center holes. Given that the hole visit activity was very different for individual animals, we also decided that this individual variance should be included into the analyses of odor effects on hole visits. Therefore, hole visits were presented as the percentage of total head dips (cf. Moy et al., 2008).

To avoid floor or ceiling effects we further excluded animals from the behavioral analysis when these rats were either too inactive (i.e., few total hole visits) or too active (i.e., many total hole visits). Based on these thoughts, the testing protocols of the following studies were designed and the exclusion criteria were defined.

## Experiment 2

It is well-established that aversive odors, such as predator odors, innately induce a variety of defensive responses including avoidance and escape behavior (Dielenberg and McGregor, 2001; Apfelbach et al., 2005; Masini et al., 2005). On the other hand, attractive odors, such as the odors of female conspecifics, are approached (Liberles, 2014). To investigate whether these behaviors can also be observed in the olfactory hole-board test for rats, we placed urine samples of carnivores, female conspecifics and a water control sample under the corner holes of the hole-board. We expected that holes with aversive odors will be avoided (i.e., less hole visits) and holes with attractive odors will be preferred (i.e., more hole visits).

### Subjects and Procedure

Fourteen male Sprague-Dawley rats were used in this experiment. The following odor samples were presented: fox urine, bobcat urine, female rat urine, and water. The locations of the odor samples were pseudo-randomized.

### Results

**FIGURE 2:**
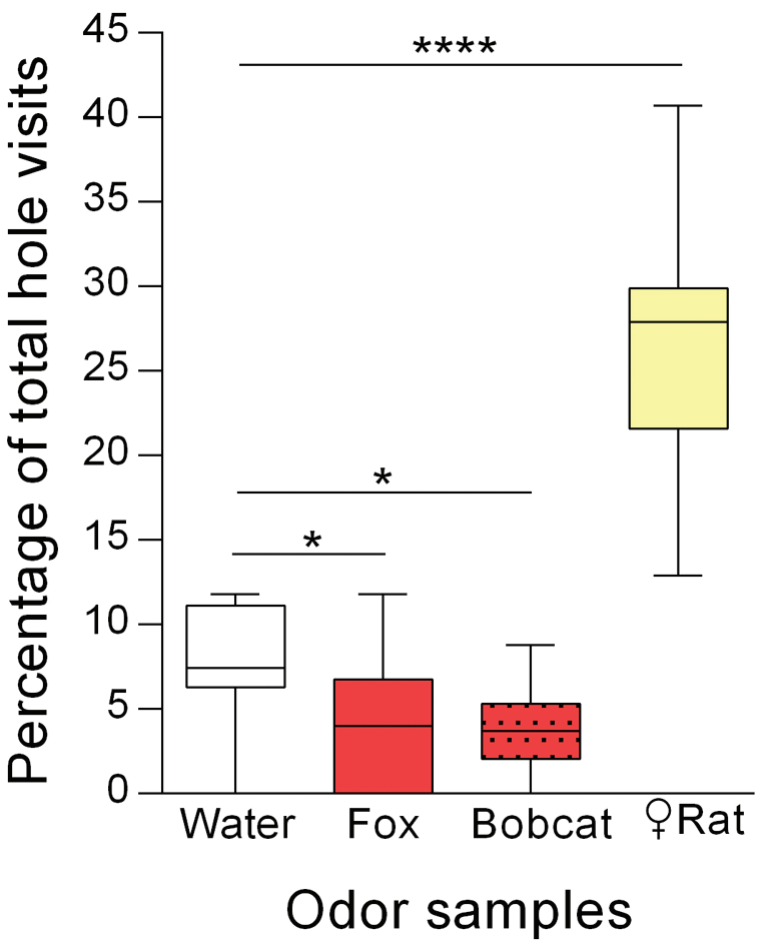
**Rats (*n* = 14) avoided holes with carnivore urine whereas holes with female rat urine were preferred.** Percentages of total hole visits (median) for the different corner holes are shown. ^∗^*p* < 0.05; ^∗∗∗∗^*p* < 0.0001 comparisons as indicated (Holm-Sidak’s multiple comparison test after significant main effects in an ANOVA).

The percentage of total hole visits for each corner hole of the present experiment is shown in **Figure 2**. The different odor samples significantly affected the corner hole visits [ANOVA: *F*_(3,39)_ = 54.85; *p* < 0.0001]. *Post hoc* pairwise comparisons with the water control indicated a strong increase of visits to the holes with female rat urine (Holm-Sidak’s test: *p* < 0.0001), while holes with fox urine (*p* = 0.03) or bobcat urine (*p* = 0.02) were visited less often. The mean number of total hole visits was 39 (data not shown).

### Discussion

*Experiment 2* investigated whether simultaneous presentation of both aversive and attractive odors led to changes in hole visit behavior. We showed that holes with carnivore urine samples were clearly visited less often than the hole with water, i.e., carnivore urine was avoided. These results support findings from previous studies showing avoidance behavior to carnivore urine. For instance, Osada et al. (2013) observed that mice similarly avoided the short arm of a Y-maze when it contained wolf urine. Using an open-field test we previously showed that rats avoid the quadrant or corner of the testing arena containing carnivore urine, e.g., from foxes, bobcats, pumas, coyotes, or lions (Fendt, 2006; Ferrero et al., 2011; Wernecke et al., 2015). This is confirmed by field studies demonstrating that carnivore urine samples (e.g., dingo, coyote, bobcat, wolf) are effective repellents protecting forestry and agricultural areas from feeding-related damage (Nolte et al., 1994; Bramley and Waas, 2001; Parsons et al., 2007).

Our second observation is that rats were attracted to the hole containing urine from female rats. Sexually naive male mice similarly preferred to investigate female urine over water in a Y-maze test (Pankevich et al., 2006). In the present experiment, the female urine sample was presented simultaneously with aversive carnivore urine samples. Since we were able to measure these appetitive effects of the female urine samples, we suggest that the different odor samples did not strongly diffuse within the hole-board testing apparatus and that avoidance/preference responses were most likely restricted to the holes containing the particular odor sample. Otherwise, an increase in general anxiety due to the recognition of aversive carnivore odors should be detectable. This would most probably reduce sexually motivated behaviors like approach to female urine samples (cf. Retana-Marquez et al., 1996; Rhees et al., 2001; Kobayashi et al., 2013).

Taken together, the present experiment is in agreement with the rats’ natural motivation to approach odors of potential mating partners (Liberles, 2014) and to avoid odors of carnivores (Apfelbach et al., 2005; Masini et al., 2005). Importantly, using the olfactory hole-board test, we are able to study olfactory avoidance and preference behavior to different types of odors presented on the same hole-board in the same test session.

## Experiment 3

The previous experiment showed that rats avoid holes with carnivore urine and preferred holes with female rat urine. However, this phenomenon could also be explained by a simple avoidance of odors from other species, whereas odors from conspecifics are preferred. To exclude this possibility, we exposed rats to urine samples from an herbivorous species (elk), an omnivorous species (mona monkey) and a carnivorous species (puma). Based on previous studies (Fendt, 2006; Ferrero et al., 2011), we would expect that carnivore but not herbivore urine samples will be avoided, whereas omnivore urine may lead to an intermediate response.

### Subjects and Procedure

Twenty two male Sprague-Dawley rats were included in this experiment. Rats were exposed to carnivore urine (Puma, *Puma concolor*), herbivore urine (Elk, *Cervus canadensis*), omnivore urine (Mona monkey, *Cercopithecus mona*) and water as a control odor.

### Results

**FIGURE 3:**
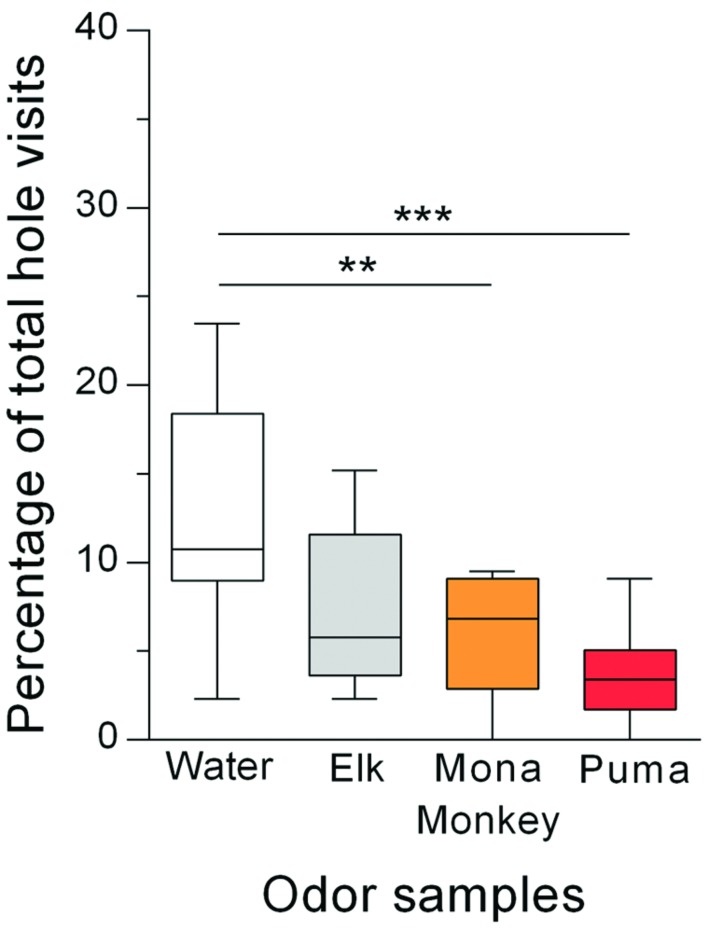
**Rats (*n* = 22) avoided holes with omnivore and carnivore urine but not holes with herbivore urine.** Percentages of total hole visits (median) for the different corner holes are shown. ^∗∗^*p* < 0.01; ^∗∗∗^*p* < 0.001 comparisons as indicated (Dunn’s multiple comparison test after significant main effects in the Friedman test).

Again, the holes with the different odor samples were differently visited by the rats (**Figure 3**), as indicated by a significant odor effect (Friedman test: *Q* = 20.98; *p* = 0.0001). *Post hoc* pairwise comparisons of the percentages of corner hole visits with the percentage of water control hole visits showed that the holes with the urine from mona monkeys and pumas were avoided [Dunn’s test: *p* = 0.009 (mona monkey urine); *p* = 0.0001 (puma urine)], whereas the holes with elk urine were not differently visited (*p* = 0.14) than the water control hole. The mean number of total hole visits was 31 (data not shown).

### Discussion

Rats were exposed to urine samples from an elk, mona monkey and puma, as representatives for herbivore, omnivore, and carnivore species, respectively. Rats avoided the holes containing urine from either the puma or the mona monkey, whereas the holes with elk urine appeared to be neutral (**Figure 3**). These findings as well as similar findings from literature (Ramp et al., 2005; Fendt, 2006; Du et al., 2012) suggest that prey animals are able to discriminate between urine of harmless herbivore species and urine of omnivore or carnivore species, both being potential predators. This would be an important evolutionary adaptation since rats would only invest energy for the defense from potential predators but would not waste energy with defensive responses to odors of herbivore species which are no threat to rats. The question now is by which mechanisms do rats innately recognize urine from potential predators? One possibility is that rats detect predators through common metabolites derived from a carnivorous diet (Nolte et al., 1994; Berton et al., 1998; Ferrero et al., 2011). Such a metabolite could be 2-phenylethylamine (PEA), a component of most carnivore species’ urine and also of some omnivore species’ urine (Ferrero et al., 2011). Only moderate concentrations of PEA have been identified in urine samples of omnivores or smaller carnivores (e.g., ferret, fox, cat, human), while higher amounts of PEA are present in urine samples of larger feline carnivores (e.g., tiger, lion, jaguar). These different PEA levels in the urine may be responsible for the intensity of the expressed avoidance behaviors.

## Experiment 4

The aim of the present experiment was to test whether anxiolytic treatments specifically modulate the avoidance of carnivore urine holes without affecting the preference of rats to female rat urine.

Benzodiazepines are highly effective anxiolytic substances in both humans and animals (Gelfuso et al., 2014). When tested in predator odor exposure tests, different benzodiazepines (e.g., midazolam) have been reported to change defensive responsiveness to cat odor leading to decreased hiding behavior and increased approach behavior (Blanchard et al., 1998; Dielenberg et al., 1999; McGregor and Dielenberg, 1999; Siviy et al., 2010). However, we observed that treatment of rats with midazolam (0, 0.19, 0.38 mg/kg) had sedative effects and strongly dose-dependently reduced the number of total hole visits (Friedman test: *Q* = 20.46; *p* < 0.0001; Supplementary Figure S1). This makes it very difficult to evaluate whether midazolam treatment affects the avoidance response to carnivore urine.

An established anxiolytic compound with only minor sedative properties is the 5-HT_1A_ receptor agonist buspirone (Kehne et al., 1988; Carli et al., 1989; Moser, 1989). Therefore, rats were treated with buspirone and the effects on olfactory hole-board performance were tested.

### Subjects and Procedure

*Experiment 4* included 14 male Sprague-Dawley rats. 20 min prior to testing, each animal was pretreated with the vehicle (saline) or the 5-HT_1A_ receptor agonist buspirone (0.1, 1 mg/kg). Injections were given intraperitoneal (i.p.) and were administered at a volume of 1 ml/kg. Each rat received each of the three treatment conditions in a pseudo-randomized order with 24 h between each test. Rats were exposed to fox urine, coyote urine, female rat urine, and water as control odor.

### Results

**FIGURE 4:**
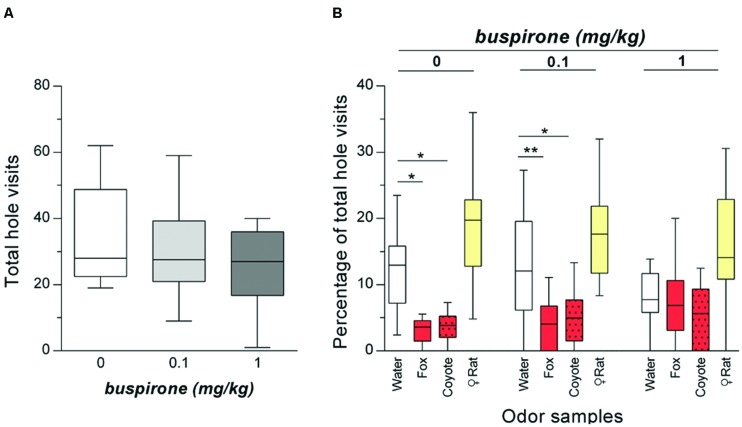
**Buspirone treatment specifically blocked the avoidance of holes with carnivore urine without sedating the rats (*n* = 14). (A)** Total number of hole visits (median) of rats after treatment with buspirone (0; 0.1; 1 mg/kg buspirone). **(B)** Percentages of total hole visits (median) for the different corner holes and for each treatment are shown. ^∗^*p* < 0.05; ^∗∗^*p* < 0.01 comparison as indicated (Holm-Sidak’s/Dunn’s multiple comparison test after significant main effects in an ANOVA/Friedman test).

The analysis of the total numbers of hole visits confirmed that buspirone has only minor sedative properties and did not significantly affect the total number of hole visits [ANOVA: *F*_(2,26)_ = 2.28; *p* = 0.129, **Figure 4A**].

The percentages of corner hole visits after treatment with saline or buspirone (0.1 mg/kg; 1 mg/kg) are illustrated in **Figure 4B**. We performed separate ANOVAs for each treatment. In saline-treated rats, there was a significant main effect of odor (Friedman test: *Q* = 29.10; *p* < 0.0001). *Post hoc* comparisons with the water control indicated that rats significantly avoided the holes with fox (Dunn’s test: *p* = 0.016) or coyote urine (*p* = 0.039). However, there was no effect of female rat urine (*p* = 0.237). After treatment with 0.1 mg/kg buspirone, there was still a significant main effect of odor [ANOVA: *F*_(3,39)_ = 14.81; *p* < 0.0001] with holes with fox urine (Holm-Sidak’s test: *p* = 0.005) or coyote urine (*p* = 0.042) being avoided. The holes with female rat urine were not visited more often by rats than the water hole (*p* = 0.105). Notably, different effects were observed after treatment with 1 mg/kg buspirone. Although there was again a main effect of odor (Friedman test: *Q* = 12.05; *p* = 0.007), the percentages of total hole visits for the different corner holes were not different from water [Dunn’s tests: *p* > 0.999 (fox urine); *p* = 0.563 (coyote urine); *p* = 0.171 (female rat urine)].

### Discussion

Treatment with the 5-HT_1A_ receptor agonist buspirone dose-dependently blocked the avoidance of holes with carnivore urine in the olfactory hole-board test. Importantly, buspirone did not affect the total hole visits and the visits of holes with attractive female rat urine (**Figures 4A,B**) indicating a specific anxiolytic effect on behavioral changes induced by carnivore urine. We further showed that the olfactory hole-board test is inappropriate for testing compounds with strong sedative effects such as midazolam (Supplementary Figure S1).

Our finding that buspirone reduced predator odor-induced defensive behavior supports previous findings showing that treatment with 8-OHDPAT [(±)-8-hydroxy-2-(di-*n*-propylamino) tetralin], another 5-HT_1A_ receptor agonist, decreases freezing and increases approach behavior to TMT (2,4,5 trimethylthiazoline), a synthetic predator odor (Shields and King, 2008). Similarly, it has been demonstrated that buspirone effectively reduces anxiety in other rodent anxiety models, such as the elevated-plus-maze test and the black/white exploration test (Moser, 1989; Hendrie et al., 1997).

## General Discussion

Rodents, as most other mammals, are predominantly olfactory oriented and largely depend on olfactory cues for operating in their environment (Sotnikov et al., 2011; Galliot et al., 2012). Therefore, odors are of considerable significance in guiding nearly every class of animal behavior (Doty, 1986) and their perception and discrimination are believed to be crucial for survival and reproduction. In this sense, the recognition of predator odors and odors of the sexual counterpart is critically important. The former induces defensive behaviors, whereas the latter induces attraction behavior in rats.

The aim of the present study was to assess whether the olfactory hole-board test can be used as a behavioral paradigm for investigating olfactory preference and avoidance to biologically relevant odors, as well as whether such a preference or avoidance can be selectively modulated by pharmacological treatments. We made use of the rats’ natural preference for corner holes in the hole-board test and examined whether this pattern of hole preference could be manipulated by placing both appetitive and aversive odors under these holes. Using the innate preference for corner holes allowed us to circumvent a floor effect since odor-induced avoidance responses are more easily detected when the holes are frequently visited under control conditions. The key advantage of the olfactory hole-board test is that it allows testing animals’ responses to four odors in a single test session. Moreover, since appetitive odor samples can be presented simultaneously with aversive odor samples under different corner holes, we were further able to test preference and avoidance responses at the same time. This is unique, since most research focused on the effects of biologically-relevant odors on the behaviors of rats used multi-trial paradigms (e.g., olfactory habituation/dishabituation task) with sequential presentations of different odors (Mandairon et al., 2009; Silverman et al., 2010; Lehmkuhl et al., 2014). Moreover, because rodents in nature are not exposed to only one pure odor but to several odors at the same time, the olfactory hole-board may present also a more natural test situation.

As mentioned in *Experiment 1*, Moy et al. (2008) had also developed an olfactory version of the hole-board test. In contrast to our study, they made use of the mice’s natural aversion of the center holes and tested whether the placement of novel, appetitive odors in these holes may modify this innate aversion. Consequently, a lack of a hole preference shift to the center holes has been interpreted to reflect the resistance to adapt their behavioral responses in regard to environmental factors (Moy et al., 2008). This suggests that the hole-board test in association with the presentation of odor samples is versatile allowing the study of multiple research issues. While the mouse olfactory hole-board was only used to test appetitive odor samples, in our version, rats were exposed to both appetitive and aversive odor samples.

The quality of the olfactory hole-board test in testing aversion and preferences in the same test session was shown by *Experiments 2* and *3*. Rats avoided visiting corner holes with urine of potential predators (fox, bobcat, puma, coyote, mona monkey). Simultaneously, rats visited holes with urine from female rats more often indicating attraction behavior. Importantly, the odor-induced avoidance response was specific to the urine of omnivore and carnivore species. In contrast, the number of visits to holes with urine of an herbivore species was indistinguishable to that of the control holes. This supports the idea that rats are able to discriminate between urine of different species, both predator and non-threatening species (Ramp et al., 2005; Fendt, 2006; Ferrero et al., 2011; Du et al., 2012). Regarding the experiments with female rat urine presentation (*Experiments 2 and 4*), a variable efficiency to induce hole preference was recognizable. It has been shown that male rats are more attracted to odors of estrous females than that of non-estrus females (Hosokawa and Chiba, 2005; Achiraman and Archunan, 2006; Achiraman et al., 2010). We collected urine regardless of the female’s estrus cycle stage. Therefore, different amounts of estrus urine in the different odor samples may serve as a likely explanation for the varying effectiveness of female rat urine to attract male rats.

The present study further tested whether the avoidance of carnivore urine holes can be reduced by treating the rats with the anxiolytic compounds midazolam or buspirone (*Experiment 4*). Treatment with buspirone specifically abolished the avoidance response to holes with carnivore urine. Notably, such effects are difficult to detect when the anxiolytic compound has strong sedative effects, as was the case with midazolam since too few hole visits were observed after midazolam treatment. The finding that the olfactory hole-board test provides direct measures of olfactory responses in rats that can be specifically pharmacologically manipulated further makes it possible to use this test to examine, for instance, the specificity of anxiolytic treatment effects.

## Conclusion

The present study demonstrates that the olfactory hole-board test may provide an appropriate tool for the assessment of olfactory aversion and preferences in rats. In contrast to many other testing paradigms, this paradigm allows testing of up to four odors simultaneously in single trials. Furthermore, the olfactory hole-board test is applicable to test anxiolytic treatments without sedating properties indicating predictive validity.

## Conflict of Interest Statement

The authors declare that the research was conducted in the absence of any commercial or financial relationships that could be construed as a potential conflict of interest.
