# Supplementary material for: The olfactory hole-board test in rats: a new paradigm to study aversion and preferences to odors
Source: Front Behav Neurosci. 2015 Aug 25;9:223. doi: 10.3389/fnbeh.2015.00223 (PMC4548150; doi:10.3389/fnbeh.2015.00223)
Supplement: Supplementary file 1 [file Data_Sheet_1.DOCX]

***Supplementary Material***

**The olfactory hole-board test: a new paradigm to study aversion and preferences to odors in rats**

Kerstin E.A. Wernecke^1,2,*^, Markus Fendt^1,2^

*^1^Institute for Pharmacology and Toxicology, Otto-von-Guericke University Magdeburg, Magdeburg, Germany, ^2^Center for Behavioral Brain Sciences, Magdeburg, Germany*

*Correspondence:

Institute for Pharmacology and Toxicology

Otto-von-Guericke University Magdeburg

Leipziger Strasse 44

D-39120 Magdeburg, Germany

[kerstin.wernecke@med.ovgu.de](mailto:kerstin.wernecke@med.ovgu.de)


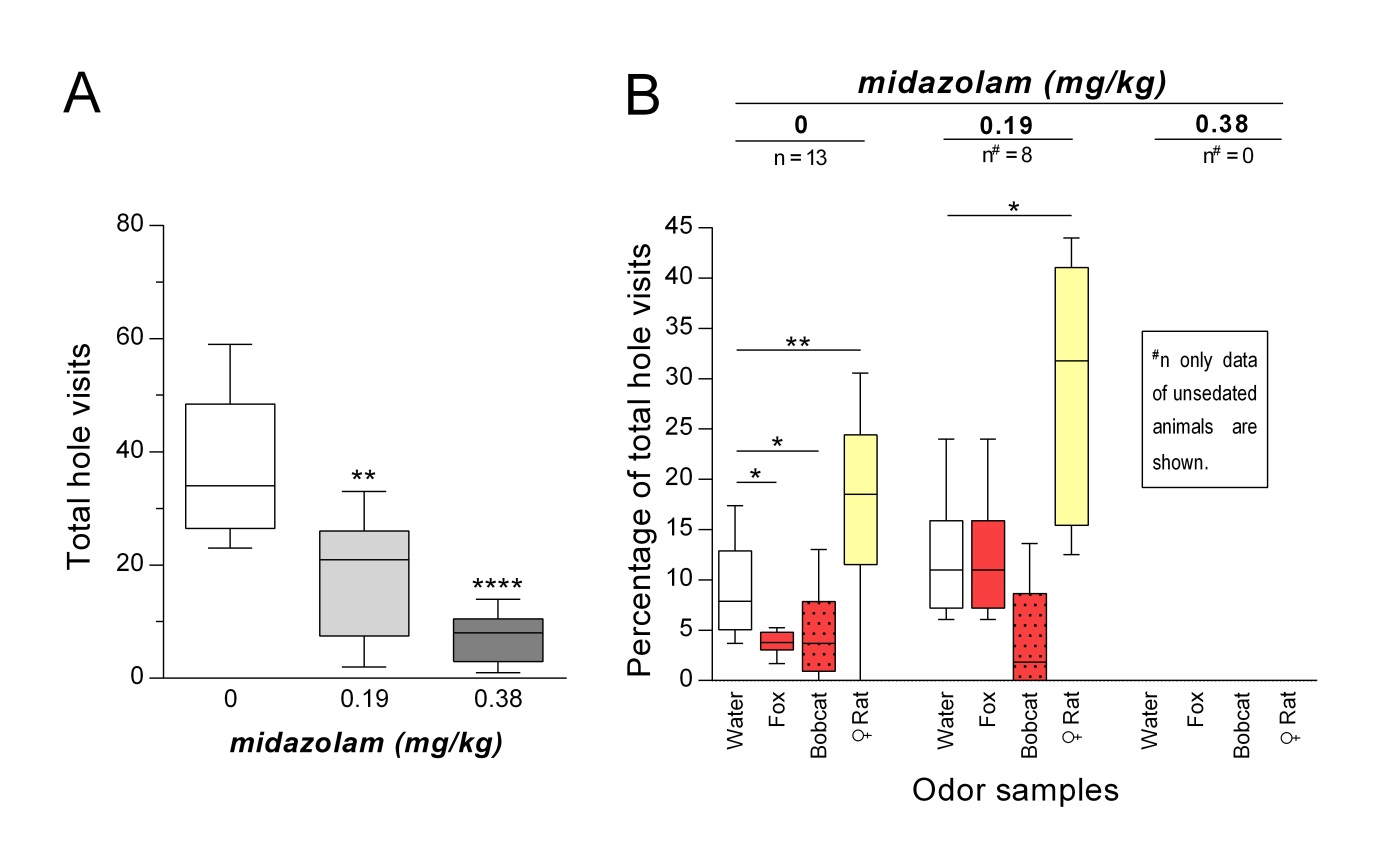


**SUPPLEMENTARY FIGURE 1 Midazolam treatment dose-dependently sedated rats (n = 13) (A)** Total number of hole visits (median) of rats after treatment with midazolam (0; 0.19; 0.38 mg/kg midazolam). **(B)** Percentages of total hole visits (median) for the different corner holes and for each treatment are shown. **p < 0.01; ****p < 0.0001 comparison to saline treatment (0 mg/kg); *p < 0.05; **p < 0.01 comparison as indicated (Holm-Sidak’s/Dunn’s multiple comparison test after significant main effects in an ANOVA/ Friedman test).
